# Adverse event reporting signals for SGLT-2 inhibitor–metformin combination therapy: a disproportionality analysis of the FAERS database

**DOI:** 10.3389/fendo.2026.1917352

**Published:** 2026-09-04

**Authors:** Wei Zhou, Yimiao Xia, Jing Li, Facai Wang

**Affiliations:** Department of Pharmacy, The Lu’an Hospital Affiliated to Anhui Medical University (The Lu’an People’s Hospital), Lu’an, China

**Keywords:** adverse drug events, FAERS database, metformin, SGLT-2 inhibitors, type 2 diabetes mellitus

## Abstract

**Objective:**

This study aimed to evaluate the real-world safety profile of combination therapy with a sodium-glucose cotransporter-2 inhibitor (SGLT-2i) and metformin relative to monotherapy, utilizing the U.S. Food and Drug Administration Adverse Event Reporting System (FAERS) database.

**Methods:**

We extracted adverse event (AE) reports from the FAERS database from the first quarter (Q1) of 2013 to the fourth quarter (Q4) of 2025 in which the combined regimen (ertugliflozin, dapagliflozin, empagliflozin, or canagliflozin plus metformin) was designated as the primary suspect. Disproportionality analyses were performed using the Reporting Odds Ratio (ROR), Proportional Reporting Ratio (PRR), Bayesian Confidence Propagation Neural Network (BCPNN), and Multi-item Gamma Poisson Shrinker (MGPS) algorithms. Additionally, we examined the time-to-onset distribution and the clinical outcomes of the documented AEs.

**Results:**

A total of 23,407 combination-therapy reports were included. At the System Organ Class (SOC) level, ROR-based signal detection identified pronounced disproportionalities in metabolism and nutrition disorders (ROR = 9.00), renal and urinary disorders (ROR = 3.91), reproductive system and breast disorders (ROR = 2.59), and infections and infestations (ROR = 2.49). At the Preferred Term (PT) level, the five most frequently reported events cross-validated by all four algorithms were diabetic ketoacidosis (n = 3,522), weight decreased (n = 1,294), blood glucose increased (n = 1,226), acute kidney injury (n = 1,153), and euglycaemic diabetic ketoacidosis (euDKA, n = 1,114). Notably, euDKA demonstrated the highest signal intensity (ROR = 335.43), with the point estimate for the combination group being numerically higher than that observed for SGLT-2i monotherapy (ROR = 185.24). Regarding documented outcomes, hospitalisation or prolonged hospitalisation constituted the most prevalent serious outcome (33.7%), followed by life-threatening events (7.6%) and death (2.7%). In the sub-cohort with definitive time-to-onset data, 39.56% of AEs occurred within the first 30 days following treatment initiation.

**Conclusion:**

This pharmacovigilance analysis of the FAERS database detected significant disproportionality signals for SGLT-2i/metformin combination therapy, particularly within metabolic/nutritional, renal/urinary, and infectious disease categories. The euDKA signal appeared prominent in descriptive comparisons. The predominance of hospitalisation-related events, coupled with the substantial concentration of AEs within the initial 30-day window, highlights the early treatment phase as a potentially critical monitoring period. These exploratory findings warrant rigorous validation through prospective cohort studies.

## 1 Introduction

The global epidemic of diabetes mellitus is accelerating at an alarming rate, with projections indicating that the patient population will surpass 1.31 billion by 2050 (1). Type 2 diabetes mellitus (T2DM), which accounts for over 90% of these cases, is pathophysiologically hallmarked by peripheral insulin resistance and progressive pancreatic β-cell dysfunction (2–4). Given the increasing prevalence, the resultant end-organ damage—encompassing major adverse cardiovascular events, nephropathy, and retinopathy—imposes a substantial clinical and socioeconomic burden, thereby rendering the pursuit of both potent and safe pharmacological interventions increasingly urgent (5–7). Owing to the progressive nature of T2DM, monotherapy frequently proves insufficient for sustaining long-term glycaemic targets, making combination regimens a cornerstone of contemporary clinical practice (8, 9). Current guidelines advocate for the addition of novel glucose-lowering agents to metformin backbone therapy. Among these, sodium-glucose cotransporter-2 inhibitors (SGLT-2i) have garnered substantial attention, primarily due to their well-documented and robust cardiovascular and renal protective effects (10, 11). Notably, emerging evidence suggests that the SGLT-2i and metformin dual regimen confers superior outcomes compared with other combinations, including a significantly reduced risk of dementia (hazard ratio [HR] = 0.62) and all-cause mortality (HR = 0.54), along with slower chronic kidney disease progression (12, 13). Despite these therapeutic benefits, the real-world safety profile of this specific dual-therapy strategy remains undercharacterised, with pharmacovigilance data remaining limited despite its increasing clinical use. To address this critical knowledge gap, we systematically mined the U.S. Food and Drug Administration Adverse Event Reporting System (FAERS) database to detect adverse event signals associated with SGLT-2i use in combination with metformin. Our objective is to furnish a comprehensive, real-world pharmacovigilance benchmark that can serve as an actionable reference for clinicians navigating the risk-benefit balance of this increasingly prevalent combination.

## 2 Methods

### 2.1 Data source and processing

This retrospective pharmacovigilance study sourced data from the U.S. Food and Drug Administration (FDA) Adverse Event Reporting System (FAERS) database, covering the period from the first quarter (Q1) of 2013 through the fourth quarter (Q4) of 2025 (https://fis.fda.gov/extensions/FPD-QDE-FAERS/FPD-QDE-FAERS.html). Each quarterly dataset comprises seven distinct yet interconnected tables—namely, demographic information (DEMO), drug information (DRUG), adverse events (REAC), therapeutic outcomes (OUTC), report sources (RPSR), therapy duration (THER), and indications (INDI)—all of which are linked via the unique PRIMARYID field. In accordance with FDA recommendations for data integrity, we applied a rigorous two-step deduplication procedure to the extracted records: first, we retained only the most recent FDA_DT entry for each unique CASE_ID; subsequently, where both CASE_ID and FDA_DT remained identical, we selected the record with the highest PRIMARYID to ensure a non-redundant and analytically robust dataset.

### 2.2 Data extraction and exposure definition

We identified adverse event reports from the FAERS database in which an SGLT-2 inhibitor (ertugliflozin, dapagliflozin, empagliflozin, or canagliflozin) and metformin were designated as primary suspect (PS) agents. The search strategy incorporated the generic terms *“ertugliflozin”*, *“dapagliflozin”*, *“empagliflozin”*, *“canagliflozin”*, and *“metformin”*, alongside their principal brand names (e.g., Farxiga^®^, Jardiance^®^, Invokana^®^; full list provided in **Supplementary Table 1**). Exposure cohorts were defined according to the following mutually exclusive criteria: (1) the combination group, comprising reports in which both an SGLT-2i and metformin were documented within the same CASE_ID; (2) the SGLT-2i monotherapy group, containing reports of any SGLT-2i without concomitant metformin; and (3) the metformin monotherapy group, containing reports of metformin without concomitant SGLT-2i. Notably, cases involving multiple distinct SGLT-2i agents within a single CASE_ID were retained and classified under the combination group, to reflect real-world prescribing complexity, including potential therapeutic interchange or overlapping supply. To maintain methodological rigour in exposure categorisation and to mitigate potential confounding introduced by fixed-dose combination (FDC) products—particularly given the incomplete brand-name reporting for FDCs in the database—all FDC formulations were explicitly excluded from the analysis. For outcome standardisation, all reported adverse events were coded according to the Medical Dictionary for Regulatory Activities (MedDRA, version 28.1), wherein individual terms were aggregated into Preferred Terms (PTs) and subsequently mapped to their corresponding System Organ Classes (SOCs).

### 2.3 Statistical analysis and signal detection

To identify potential associations between the studied regimens and adverse events, we performed disproportionality analysis—a well-established pharmacovigilance methodology extensively employed for postmarketing safety surveillance (14). The analytical framework was based on a standard 2 × 2 contingency table (**Table 1**), and signal detection was conducted using four complementary algorithms: the Reporting Odds Ratio (ROR), the Proportional Reporting Ratio (PRR), the Bayesian Confidence Propagation Neural Network (BCPNN), and the Multi-item Gamma Poisson Shrinker (MGPS) (15–18). ROR and PRR, as frequentist approaches, are characterised by high sensitivity. Specifically, ROR offers enhanced control over reporting bias in scenarios with low event counts, whereas PRR exhibits higher tolerance for underreporting of adverse events (16, 19). Nevertheless, the stability of both estimators may be compromised when the number of target events is extremely limited (20). In contrast, the Bayesian algorithms—BCPNN and MGPS—provide superior specificity, signal robustness, and a diminished risk of false-positive findings. BCPNN is particularly adept at integrating heterogeneous data sources to facilitate cross-validation, while MGPS demonstrates unique advantages in detecting signals for rare adverse events (17, 21). By collectively applying these four distinct yet complementary methods, we sought to achieve a balanced trade-off between sensitivity and specificity, thereby improving the reliability and interpretability of the detected safety signals.

**Table 1:** Fourfold table of ratio imbalance measurement methods.

Drug	Number of target adverse event reports	Number of other adverse event reports	Total
Target drug	a	b	a + b
Other drugs	c	d	c + d
Total	a + c	b + d	N

N = a + b + c + d.

We adopted a tiered signal detection strategy, applying distinct analytical frameworks at the Preferred Term (PT) and System Organ Class (SOC) levels. The PT-level analysis served as the primary investigative tier; to rigorously control for false-positive risk, a positive signal was declared only when a given PT simultaneously satisfied all four prespecified thresholds, as detailed in **Table 2**. Conversely, the SOC-level analysis was designed to provide a descriptive, system-wide overview of the primary organ systems or disease categories affected. For this purpose, we employed the ROR method exclusively for the descriptive ranking of signal intensity. A signal at the SOC level was considered detectable by the ROR algorithm only when the total number of reports for that SOC was ≥ 3 and the lower bound of the 95% confidence interval for the ROR exceeded 1. Importantly, these SOC-level findings were interpreted solely as a macro-level contextual mapping of the PT-derived signals and were not utilised as evidence for establishing causal associations.

**Table 2:** Calculation formulas and standards for signal detection.

Method	Formula	Threshold
ROR	ROR = (a/c)/(b/d), 95% CI = e^ln(ROR) ± 1.96(1/a + 1/b + 1/c + 1/d)^0.5^	95% CI > 1, a ≥ 3
PRR	PRR = [a/(a + b)]/[c/(c + d)], χ^2^ = (|ad − bc|−n/2)2n/[(a + b)(c + d)(a + c)(b + d)]	PRR ≥ 2, *χ*2 ≥ 4, a ≥ 3
MGPS	EBGM = a(a + b + c + d)/[(a + c)(a + b)], EBGM05 = e^ln(EBGM) − 1.64(1/a + 1/b + 1/c + 1/d)^0.5^	EBGM05 > 2, a > 0
BCPNN	IC = log_2_a(a + b + c + d)/[(a + c)(a + b)], IC025 = e^ln(IC) − 1.96(1/a + 1/b + 1/c + 1/d)^0.5^	IC025 > 0

EBGM, empirical Bayesian geometric mean; IC, information component.

To mitigate the interpretive challenges arising from excessive granularity and potential fragmentation inherent to the MedDRA Preferred Term (PT) hierarchy, we conducted a supplementary aggregated analysis at the clinically relevant event level, in addition to the primary PT-level evaluation. Specifically, we categorised events into three composite groups—namely, ketoacidosis events, genital infections, and renal injury events—following which we recalculated the cumulative reporting counts and derived pooled ROR estimates with corresponding 95% confidence intervals for each category. This approach was designed to furnish safety signal assessments that are more directly interpretable within the context of real-world clinical reasoning and practice. In parallel, all primary disproportionality analyses were performed using the full FAERS database as the background comparator to detect adverse event signals specifically associated with the SGLT-2i and metformin combination. To evaluate the potential influence of concomitant or alternative glucose-lowering agents on the stability and robustness of our findings, we additionally implemented a prespecified sensitivity analysis. In this sensitivity framework, the background comparator population was restricted exclusively to reports involving other antidiabetic medications—explicitly excluding both SGLT-2 inhibitors and metformin—thereby enabling a more isolated assessment of the combination regimen’s safety profile against a therapeutically relevant control backdrop.

### 2.4 Time-to-onset analysis

The time to onset of adverse events (AEs) was defined as the interval between the treatment initiation date (START_DT, extracted from the THER table) and the date of the AE occurrence (EVENT_DT, sourced from the DEMO table). Before the analysis, we excluded records with data entry errors in which the event date preceded the treatment start date (i.e., EVENT_DT < START_DT), and those with inaccurate dates or duplicate entries, to ensure chronological integrity and data quality. Given the inherent right-censoring of the time-to-event data and the potential for non-constant hazard trajectories over the treatment course, we fitted the time-to-onset distributions for the three cohorts using a Weibull parametric survival model. This model characterises the survival process by simultaneously estimating a shape parameter (*k*) and a scale parameter (*λ*). The corresponding survival function *S*(*t*) and hazard function h(*t*) are expressed as:


$$\begin{array}{c} S(t)=\exp(-\lambda t^{k})\\ h(t)=k\lambda t^{k-1} \end{array}$$

where, *t* denotes the follow-up time. The shape parameter *k* governs the directional trajectory of the hazard over time: a value of *k* > 1, with the lower bound of its 95% confidence interval exceeding 1, indicates an increasing risk of AEs with prolonged treatment exposure; a *k* equal to 1, with the 95% CI encompassing 1, suggests a constant hazard over time, consistent with an exponential decay-free process; conversely, a *k* < 1, with the upper bound of its 95% CI remaining below 1, points to a diminishing risk of AEs as treatment duration extends.

## 3 Results

### 3.1 Descriptive overview of the study population

**Figure 1:**
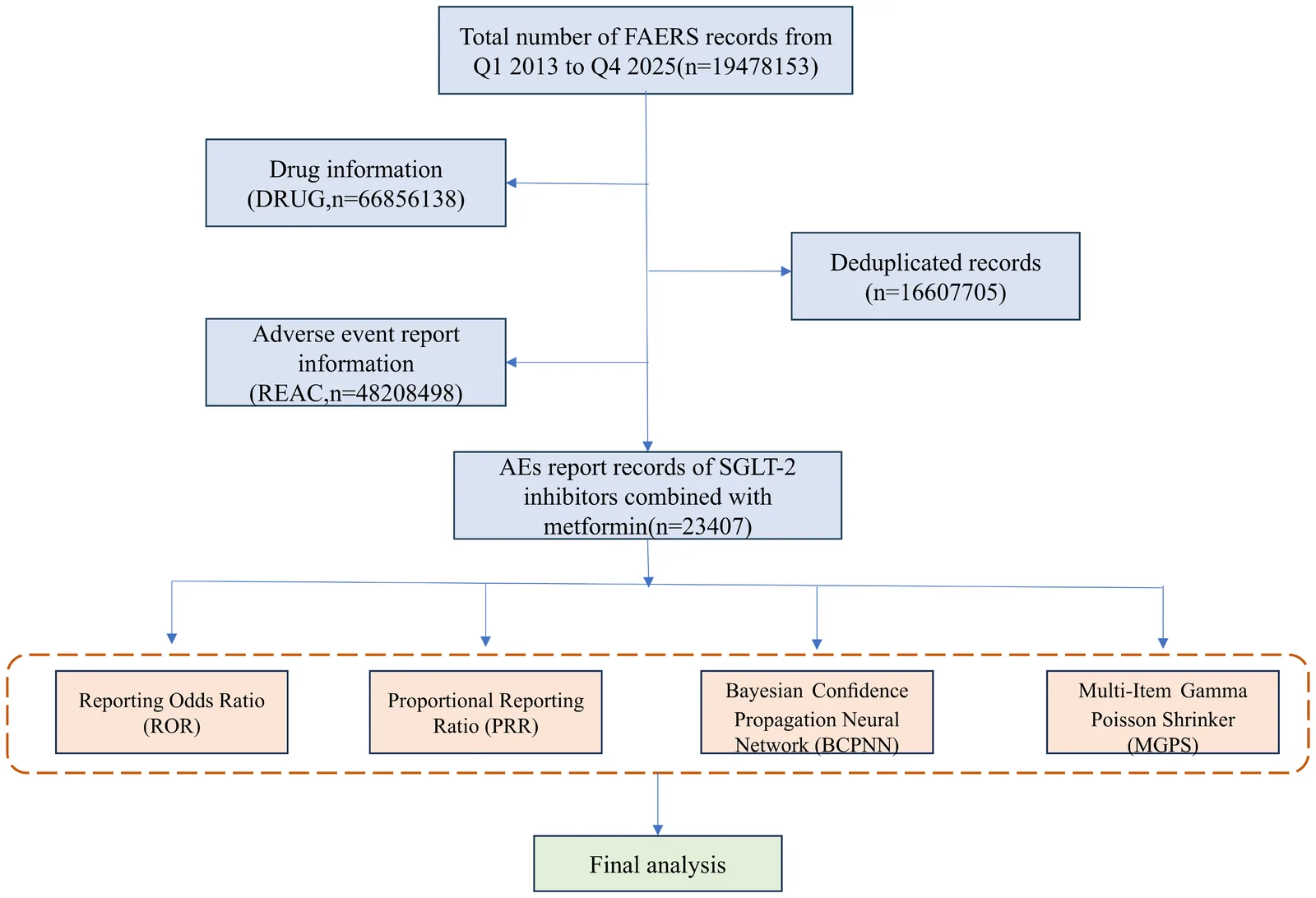
Study flow diagram (FAERS data screening and cleaning process).

**Figure 2:**
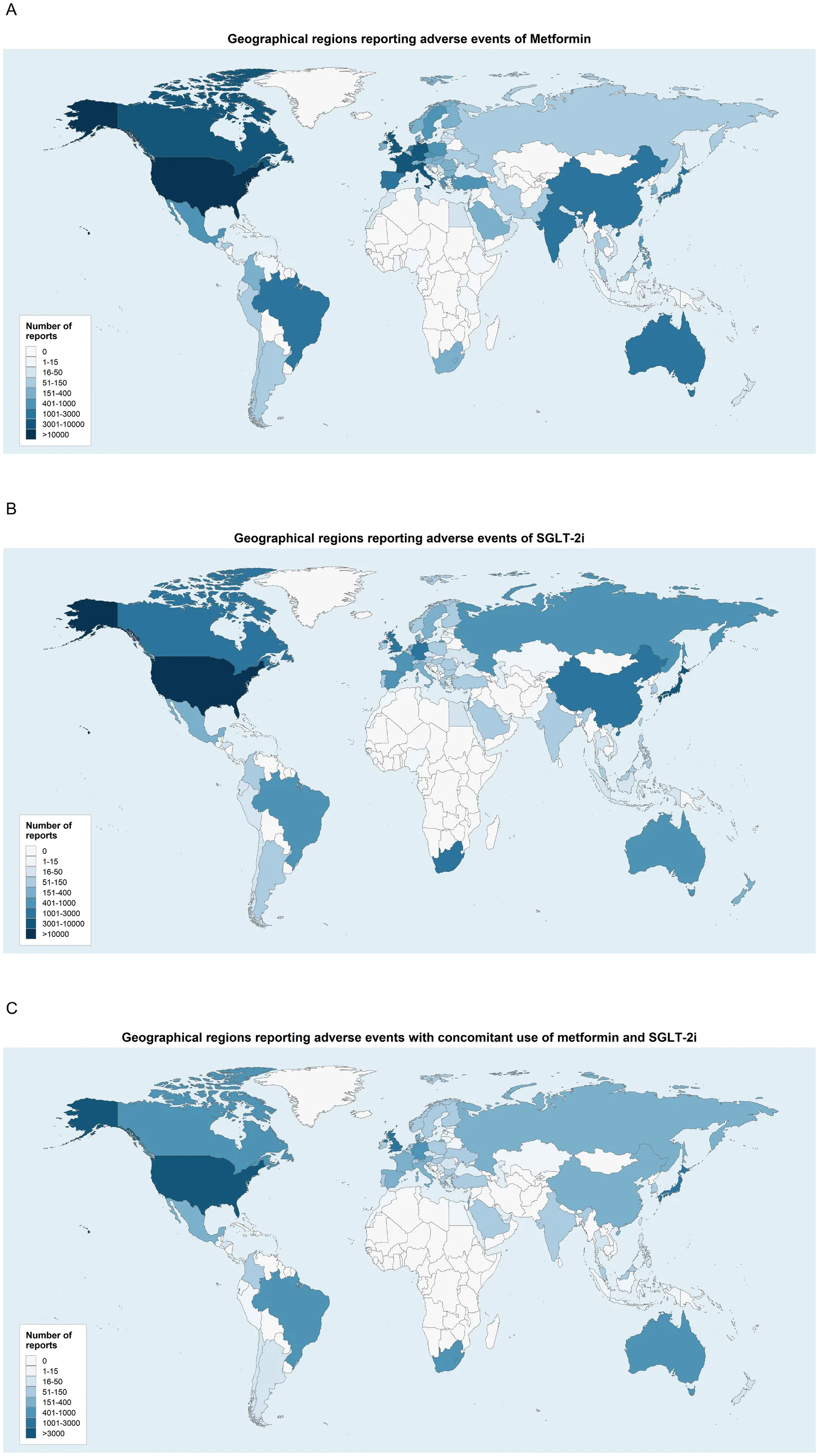
Distribution of primary reporting countries [**(A)** Metformin monotherapy; **(B)** SGLT-2i monotherapy; **(C)** combination therapy].

Between the first quarter of 2013 and the fourth quarter of 2025, a total of 19,478,153 individual records were extracted from the FAERS database. Following the prespecified deduplication and inclusion procedures, we identified 23,407 adverse event (AE) reports involving the concomitant use of an SGLT-2 inhibitor and metformin (**Figure 1**). The demographic and clinical characteristics of this cohort—including sex, body weight, age, reporter occupation, reporting year, and geographic region—are summarised in **Table 3**. Among reports for the combination regimen, there was a marginal male predominance (51.3%); however, substantial proportions of missing data were observed for body weight (60.8%) and age (23.9%), and accordingly, these demographic variables should be interpreted with appropriate caution. Regarding reporting sources, physicians (38.7%) and consumers (33.6%) constituted the two primary reporter categories. Temporally, the annual number of AE reports for the combination therapy exhibited a steady upward trend from 2013, reaching a peak of 2,903 reports in 2018, followed by a gradual decline, though numbers remained relatively elevated through to 2025. Geographically, reporting was heavily concentrated in the United States, which accounted for 52.5% of all combination-related reports, followed in descending order by the United Kingdom (8.0%), Japan (5.1%), Canada (4.2%), and Germany (3.3%) (**Figure 2**).

**Table 3:** Demographic and clinical characteristics of the study population.

Characteristics	SGLT-2i	Metformin	SGLT-2i + metformin
	(N = 74,537)	(N = 80,383)	(N = 23,407)
Sex, n (%)			
Male	33,392 (44.8%)	32,192 (40.0%)	12,004 (51.3%)
Female	29,746 (39.9%)	37,262 (46.4%)	10,410 (44.5%)
Missing	11,399 (15.3%)	10,929 (13.6%)	993 (4.2%)
Weight, n (%)			
<50 kg	393 (0.5%)	1,008 (1.3%)	184 (0.8%)
>100 kg	2,916 (3.9%)	3,636 (4.5%)	2,496 (10.7%)
50–100 kg	8,366 (11.2%)	14,269 (17.8%)	6,487 (27.7%)
Missing	62,862 (84.3%)	61,470 (76.5%)	14,240 (60.8%)
Age, years, n (%)			
<2	34 (<0.0%)	267 (0.3%)	13 (0.1%)
2–11	26 (<0.0%)	124 (0.2%)	5 (<0.0%)
12–17	42 (0.1%)	740 (0.9%)	9 (<0.0%)
18–64	17,520 (23.5%)	25,425 (31.6%)	11,218 (47.9%)
65–85	16,123 (21.6%)	24,846 (30.9%)	6,374 (27.2%)
≥85	1,786 (2.4%)	2,132 (2.7%)	199 (0.9%)
Missing	39,006 (52.3%)	26,849 (33.4%)	5,589 (23.9%)
Occupation, n (%)			
CN	30,767 (41.3%)	23,776 (29.6%)	7,863 (33.6%)
HP	4,287 (5.7%)	11,428 (14.2%)	1,094 (4.7%)
LW	79 (0.1%)	129 (0.2%)	81 (0.3%)
MD	23,104 (31.0%)	22,824 (28.3%)	9,056 (38.7%)
OT	3,691 (5.0%)	11,647 (14.5%)	1,716 (7.4%)
PH	3,902 (5.2%)	6,971 (8.7%)	1,764 (7.5%)
Missing	8,707 (11.7%)	3,608 (4.5%)	1,833 (7.8%)
Reported year, n (%)			
2013	170 (0.2%)	4,064 (5.1%)	52 (0.2%)
2014	1,416 (1.9%)	4,921 (6.1%)	557 (2.4%)
2015	7,236 (9.7%)	4,750 (5.9%)	2,866 (12.2%)
2016	4,311 (5.8%)	6,308 (7.8%)	2,330 (10.0%)
2017	4,904 (6.6%)	5,370 (6.7%)	2,477 (10.6%)
2018	6,649 (8.9%)	8,407 (10.5%)	2,903 (12.4%)
2019	5,753 (7.7%)	8,203 (10.2%)	2,573 (11.0%)
2020	4,445 (6.0%)	8,374 (10.4%)	1,996 (8.5%)
2021	4,874 (6.5%)	6,463 (8.0%)	1,816 (7.8%)
2022	6,447 (8.6%)	5,912 (7.4%)	1,428 (6.1%)
2023	8,865 (11.9%)	6,431 (8.0%)	1,407 (6.0%)
2024	8,594 (11.6%)	5,827 (7.2%)	1,450 (6.2%)
2025	10,873 (14.6%)	5,353 (6.7%)	1,552 (6.6%)
Reported countries (top five), n (%)	SGLT-2i	Metformin	SGLT-2i+Metformin
United States	54,652 (73.3%)	29,335 (36.5%)	12,277 (52.5%)
United Kingdom	2,173 (2.9%)	5,330 (6.6%)	1,875 (8.0%)
Japan	3,210 (4.3%)		1,204 (5.1%)
Canada			985 (4.2%)
Germany	1,582 (2.1%)	4,189 (5.2%)	779 (3.3%)
China	1,496 (2.0%)		
France		8,100 (10.1%)	
Italy		5,493 (6.8%)	

### 3.2 SOC-level signal detection

**Figure 3:**
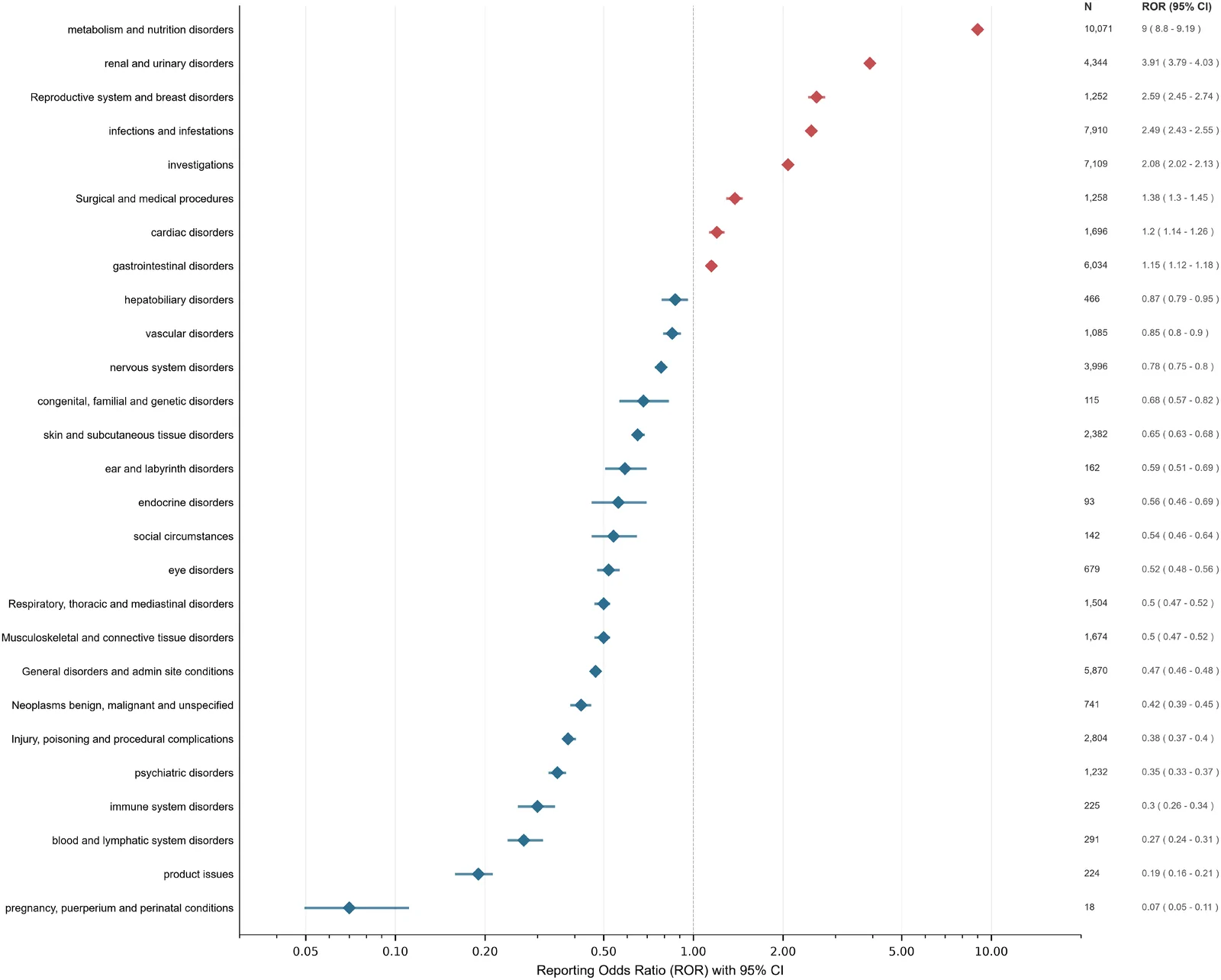
Signal strength of adverse events by System Organ Class (SOC) for combination therapy (ROR and 95% CI).

Application of the disproportionality analysis at the System Organ Class (SOC) level identified adverse drug reactions associated with the SGLT-2i and metformin combination across 27 distinct SOC categories (**Figure 3**). Utilizing the ROR method with thresholds of a minimum of three reports and a lower bound of the 95% confidence interval exceeding 1, we detected signals in eight SOCs at this hierarchical level. Of these, two—”Investigations” and “Surgical and medical procedures”—were excluded *a priori*, as they represent diagnostic or procedural categories rather than discrete clinical disease entities. The remaining six SOCs with detectable signals, ranked in descending order of ROR point estimates, were as follows: *metabolism and nutrition disorders* (n = 10,071; ROR = 9.00, 95% CI: 8.80–9.19), *renal and urinary disorders* (n = 4,344; ROR = 3.91, 95% CI: 3.79–4.03), *reproductive system and breast disorders* (n = 1,252; ROR = 2.59, 95% CI: 2.45–2.74), *infections and infestations* (n = 7,910; ROR = 2.49, 95% CI: 2.43–2.55), *cardiac disorders* (n = 1,696; ROR = 1.20, 95% CI: 1.14–1.26), and *gastrointestinal disorders* (n = 6,034; ROR = 1.15, 95% CI: 1.12–1.18). Notably, the ROR estimates for cardiac and gastrointestinal disorders were only marginally above the detection threshold, bordering on unity, indicating comparatively weak signal intensities. Consequently, these SOC-level findings should be interpreted with caution and considered solely as a contextual framework for the PT-level signals; they should not, in isolation, be construed as independent safety alerts without corroboration from the primary PT-level positive signals.

**Figure 4:**
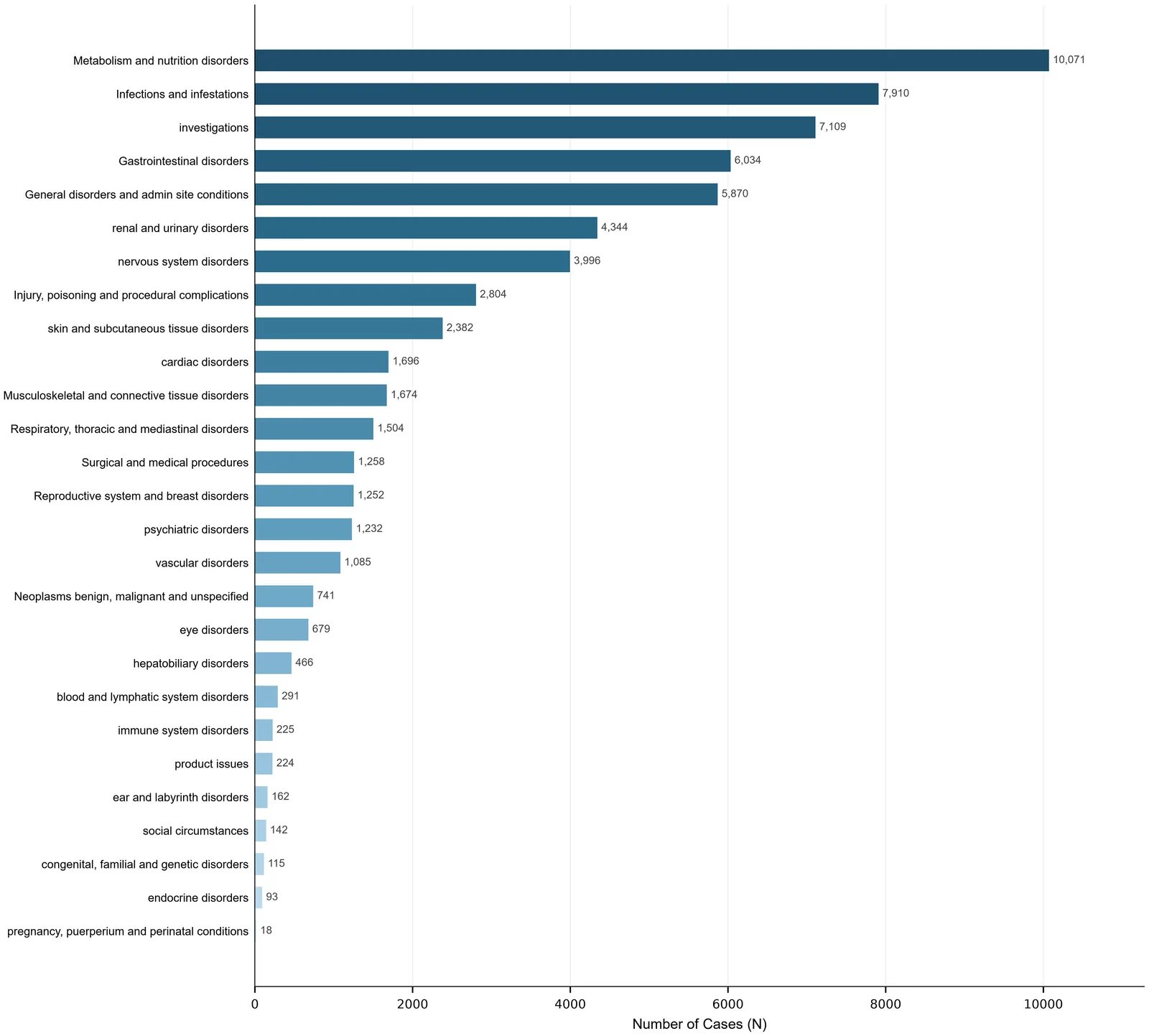
Ranking of the number of adverse event reports by 27 System Organ Classes (SOCs) for combination therapy.

Complementing the SOC-level signal intensity rankings, **Figure 4** illustrates the overlap between reporting frequency and positive ROR signals. Among the five SOCs with the highest report counts, three—metabolism and nutrition disorders, infections and infestations, and gastrointestinal disorders—also exhibited positive ROR signals. Notably, although investigations ranked third by reporting volume, this category primarily comprises laboratory or diagnostic findings rather than clinical disease entities and was therefore not considered clinically interpretable in this context. In parallel, general disorders and administration site conditions was among the most frequently reported SOCs; however, the corresponding ROR estimate did not reach statistical significance, suggesting that the observed disproportionality in this category may be influenced by reporting artefacts, detection bias, or unmeasured confounding, warranting further scrutiny in subsequent analyses.

### 3.3 PT-level signal detection

At the Preferred Term (PT) level, we performed a stringent signal detection procedure in which a positive signal was defined as one that simultaneously satisfied the prespecified thresholds for all four algorithms—ROR, PRR, BCPNN, and MGPS—after systematically excluding PTs that were unequivocally unrelated to adverse drug reactions (e.g., procedural or social history terms). Applying this rigorous framework, we evaluated the safety profiles across the three exposure cohorts—SGLT-2i monotherapy, metformin monotherapy, and the SGLT-2i/metformin combination—and ranked the detected PTs by reporting frequency, with the top 30 PTs for each cohort presented in detail (**Tables 4**–**6**). For the SGLT-2i monotherapy cohort, the five most frequently reported PTs were as follows: *death* (n = 7,376; ROR = 3.43, 95% CI: 3.35–3.51), *diabetic ketoacidosis* (n = 5,772; ROR = 136.67, 95% CI: 132.47–141.00), *fungal infection* (n = 4,020; ROR = 56.59, 95% CI: 54.71–58.55), *urinary tract infection* (n = 2,726; ROR = 6.47, 95% CI: 6.23–6.72), and *weight decreased* (n = 2,563; ROR = 3.82, 95% CI: 3.67–3.97). For the metformin monotherapy cohort, the five most frequently reported PTs were as follows: *lactic acidosis* (n = 15,498; ROR = 306.49, 95% CI: 298.72–314.46), *acute kidney injury* (n = 8,591; ROR = 11.90, 95% CI: 11.64–12.17), *diarrhoea* (n = 6,186; ROR = 2.22, 95% CI: 2.16–2.27), *metabolic acidosis* (n = 4,738; ROR = 45.73, 95% CI: 44.29–47.22), and *hypoglycaemia* (n = 4,157; ROR = 26.29, 95% CI: 25.44–27.16). For the combination (SGLT-2i + metformin) cohort, the five most frequently reported PTs were as follows: *diabetic ketoacidosis* (n = 3,522; ROR = 175.71, 95% CI: 169.27–182.40), *weight decreased* (n = 1,294; ROR = 4.63, 95% CI: 4.39–4.90), *blood glucose increased* (n = 1,226; ROR = 6.82, 95% CI: 6.44–7.22), *acute kidney injury* (n = 1,153; ROR = 6.19, 95% CI: 5.84–6.56), and *euglycaemic diabetic ketoacidosis* (euDKA; n = 1,114; ROR = 335.43, 95% CI: 312.47–360.08). Among the top 30 PTs for the combination cohort, the SOC distribution was dominated by six primary categories: *metabolism and nutrition disorders* (n = 7,865, 43.27%), *infections and infestations* (n = 3,915, 21.54%), *investigations* (n = 3,725, 20.49%), *renal and urinary disorders* (n = 2,123, 11.68%), *gastrointestinal disorders* (n = 291, 1.60%), and *general disorders and administration site conditions* (n = 259, 1.42%).

**Table 4:** Top 30 Preferred Terms (PTs) of adverse event signals associated with SGLT-2 inhibitor monotherapy.

PT	n	ROR (95% CI)	PRR (χ²)	EBGM (EBGM05)	IC (IC025)
death	7,376	3.43 (3.35–3.51)	3.31 (11,952.13)	3.29 (3.21)	1.72 (1.68)
diabetic ketoacidosis	5,772	136.67 (132.47–141)	131.6 (525,787.66)	92.75 (89.9)	6.54 (6.47)
fungal infection	4,020	56.59 (54.71–58.55)	55.15 (181,621.13)	46.99 (45.42)	5.55 (5.49)
urinary tract infection	2,726	6.47 (6.23–6.72)	6.37 (12,136.41)	6.27 (6.03)	2.65 (2.59)
weight decreased	2,563	3.82 (3.67–3.97)	3.77 (5,179.13)	3.74 (3.59)	1.9 (1.84)
blood glucose increased	2,452	5.69 (5.46–5.92)	5.61 (9,161.64)	5.53 (5.31)	2.47 (2.41)
acute kidney injury	1,942	4.32 (4.13–4.52)	4.28 (4,834.93)	4.24 (4.05)	2.08 (2.01)
osteomyelitis	1,936	55.02 (52.41–57.76)	54.35 (86,312.62)	46.41 (44.21)	5.54 (5.43)
ketoacidosis	1,845	161.53 (152.67–170.89)	159.61 (192,161.08)	105.8 (100)	6.73 (6.57)
dehydration	1,534	5.12 (4.87–5.38)	5.08 (4,951.83)	5.01 (4.76)	2.33 (2.25)
pollakiuria	1,437	14.98 (14.21–15.8)	14.85 (17,730.16)	14.22 (13.48)	3.83 (3.74)
cerebrovascular accident	1,369	3.84 (3.64–4.06)	3.82 (2,821.36)	3.79 (3.59)	1.92 (1.84)
euglycaemic diabetic ketoacidosis	1,354	185.24 (173.17–198.15)	183.62 (154,608.1)	115.8 (108.26)	6.86 (6.65)
fournier’s gangrene	1,192	356.96 (328.47–387.92)	354.21 (196,236.11)	166.09 (152.83)	7.38 (7.08)
myocardial infarction	1,170	3.57 (3.37–3.78)	3.55 (2,125.44)	3.52 (3.33)	1.82 (1.73)
renal failure	1,113	3.65 (3.44–3.87)	3.63 (2,098.4)	3.6 (3.39)	1.85 (1.76)
gangrene	1,083	100.86 (94.16–108.03)	100.16 (80,413.51)	75.99 (70.95)	6.25 (6.05)
cellulitis	1,004	8.4 (7.89–8.95)	8.35 (6,333.06)	8.16 (7.66)	3.03 (2.93)
sepsis	980	3.73 (3.5–3.97)	3.71 (1,924.09)	3.68 (3.46)	1.88 (1.78)
glycosylated haemoglobin increased	935	14.71 (13.78–15.71)	14.63 (11,345.42)	14.02 (13.13)	3.81 (3.69)
cardiac failure	896	4.75 (4.45–5.08)	4.73 (2,599.64)	4.67 (4.38)	2.22 (2.12)
renal impairment	765	3.69 (3.43–3.96)	3.67 (1,473.87)	3.64 (3.39)	1.87 (1.76)
diabetic foot infection	740	411.24 (368.48–458.96)	409.28 (130,095.2)	177.23 (158.8)	7.47 (7.03)
diabetic foot	734	123.64 (113.49–134.69)	123.06 (63,662.82)	88.44 (81.18)	6.47 (6.18)
skin ulcer	629	10.26 (9.48–11.11)	10.22 (5,068.8)	9.93 (9.17)	3.31 (3.17)
pancreatitis	610	5.63 (5.19–6.1)	5.61 (2,271)	5.53 (5.1)	2.47 (2.34)
glomerular filtration rate decreased	599	20.14 (18.54–21.88)	20.07 (10,195.11)	18.91 (17.41)	4.24 (4.08)
blood glucose abnormal	537	11.15 (10.23–12.15)	11.11 (4,772.52)	10.76 (9.87)	3.43 (3.28)
metabolic acidosis	529	7.1 (6.51–7.74)	7.08 (2,699.49)	6.94 (6.37)	2.79 (2.65)
dysuria	527	6.18 (5.66–6.73)	6.16 (2,233.72)	6.06 (5.56)	2.6 (2.46)

**Table 5:** Top 30 Preferred Terms (PTs) of adverse event signals associated with metformin monotherapy.

PT	n	ROR (95% CI)	PRR (χ²)	EBGM (EBGM05)	IC (IC025)
lactic acidosis	15,498	306.49 (298.72–314.46)	288.66 (1,710,063.91)	111.67 (108.83)	6.8 (6.76)
acute kidney injury	8,591	11.9 (11.64–12.17)	11.55 (78,013.42)	10.91 (10.67)	3.45 (3.41)
diarrhoea	6,186	2.22 (2.16–2.27)	2.19 (3,983.49)	2.17 (2.12)	1.12 (1.08)
metabolic acidosis	4,738	45.73 (44.29–47.22)	44.93 (163,037.19)	36.18 (35.04)	5.18 (5.12)
hypoglycaemia	4,157	26.29 (25.44–27.16)	25.89 (87,062.2)	22.77 (22.04)	4.51 (4.45)
blood glucose increased	3,813	5.18 (5.02–5.35)	5.12 (12,332.62)	5.01 (4.85)	2.32 (2.27)
hypotension	2,211	2.72 (2.61–2.84)	2.71 (2,353.57)	2.68 (2.57)	1.42 (1.36)
hyperkalaemia	2,001	15.84 (15.13–16.58)	15.73 (25,400.69)	14.55 (13.9)	3.86 (3.79)
renal failure	1,923	3.69 (3.53–3.86)	3.67 (3,669.72)	3.62 (3.46)	1.86 (1.79)
shock	1,500	19.38 (18.37–20.44)	19.27 (23,485.93)	17.51 (16.6)	4.13 (4.04)
dehydration	1,381	2.66 (2.52–2.8)	2.65 (1,400.82)	2.63 (2.49)	1.39 (1.31)
diabetic ketoacidosis	1,375	13.83 (13.09–14.61)	13.76 (15,128.42)	12.86 (12.17)	3.68 (3.59)
hyperglycaemia	1,323	10.13 (9.58–10.71)	10.08 (10,257.41)	9.6 (9.08)	3.26 (3.17)
glycosylated haemoglobin increased	1,289	11.98 (11.32–12.67)	11.93 (12,107.1)	11.25 (10.63)	3.49 (3.4)
renal impairment	1,198	3.37 (3.19–3.57)	3.36 (1,956.33)	3.32 (3.14)	1.73 (1.64)
cardiac arrest	1,139	3.79 (3.58–4.02)	3.78 (2,286.34)	3.73 (3.51)	1.9 (1.81)
gastrointestinal disorder	890	2.34 (2.19–2.5)	2.34 (672.96)	2.32 (2.17)	1.21 (1.11)
blood creatinine increased	881	3.64 (3.4–3.89)	3.63 (1,646.55)	3.58 (3.35)	1.84 (1.74)
pancreatitis	819	4.41 (4.11–4.73)	4.4 (2,102.12)	4.32 (4.03)	2.11 (2)
ketoacidosis	743	28.64 (26.5–30.95)	28.56 (17,063.73)	24.8 (22.95)	4.63 (4.47)
diabetes mellitus	743	2.68 (2.5–2.88)	2.68 (770.72)	2.65 (2.47)	1.41 (1.3)
multiple organ dysfunction syndrome	741	5.47 (5.08–5.88)	5.46 (2,619.17)	5.33 (4.95)	2.41 (2.3)
blood glucose decreased	738	4.08 (3.79–4.39)	4.07 (1,671.7)	4 (3.72)	2 (1.89)
hyperlactacidaemia	728	102.13 (93.25–111.86)	101.86 (46,476.34)	65.47 (59.78)	6.03 (5.79)
anuria	650	23.29 (21.47–25.28)	23.24 (12,257.3)	20.7 (19.08)	4.37 (4.21)
pancreatitis acute	631	7.58 (7–8.21)	7.57 (3,452.11)	7.3 (6.74)	2.87 (2.74)
blood glucose abnormal	624	7.56 (6.97–8.19)	7.54 (3,399.65)	7.28 (6.72)	2.86 (2.73)
hypothermia	605	16.1 (14.81–17.5)	16.06 (7,849.59)	14.83 (13.65)	3.89 (3.74)
pemphigoid	550	18.15 (16.63–19.82)	18.12 (8,084.65)	16.56 (15.17)	4.05 (3.88)
blood lactic acid increased	533	28.5 (26.01–31.23)	28.44 (12,193.09)	24.71 (22.55)	4.63 (4.43)

**Table 6:** Top 30 Preferred Terms (PTs) of adverse event signals associated with SGLT-2 inhibitor–metformin combination therapy.

PT	n	ROR (95% CI)	PRR (χ²)	EBGM (EBGM05)	IC (IC025)
diabetic ketoacidosis	3,522	175.71 (169.27–182.4)	166.14 (473,376.1)	136.16 (131.17)	7.09 (6.98)
weight decreased	1,294	4.63 (4.39–4.9)	4.56 (3,592.69)	4.54 (4.3)	2.18 (2.1)
blood glucose increased	1,226	6.82 (6.44–7.22)	6.71 (5,917.1)	6.66 (6.29)	2.73 (2.64)
acute kidney injury	1,153	6.19 (5.84–6.56)	6.1 (4,885.86)	6.05 (5.71)	2.6 (2.51)
euglycaemic diabetic ketoacidosis	1,114	335.43 (312.47–360.08)	329.63 (253,488.3)	229.22 (213.53)	7.84 (7.47)
ketoacidosis	990	169.35 (158.01–181.5)	166.76 (133,433.88)	136.58 (127.43)	7.09 (6.81)
urinary tract infection	850	4.77 (4.46–5.11)	4.72 (2,486.5)	4.7 (4.39)	2.23 (2.13)
fungal infection	814	23.87 (22.25–25.61)	23.58 (17,072.86)	22.89 (21.34)	4.52 (4.38)
dehydration	679	5.41 (5.02–5.84)	5.37 (2,401.11)	5.34 (4.95)	2.42 (2.3)
pollakiuria	576	14.07 (12.95–15.28)	13.95 (6,802.52)	13.71 (12.62)	3.78 (3.62)
fournier’s gangrene	556	249.81 (226.9–275.04)	247.66 (102,661.07)	186.38 (169.29)	7.54 (6.99)
glycosylated haemoglobin increased	548	20.44 (18.77–22.26)	20.28 (9,781.49)	19.77 (18.15)	4.31 (4.13)
metabolic acidosis	517	16.79 (15.38–18.32)	16.66 (7,447.73)	16.32 (14.95)	4.03 (3.86)
sepsis	459	4.19 (3.82–4.59)	4.17 (1,100.43)	4.15 (3.78)	2.05 (1.91)
osteomyelitis	379	22.61 (20.41–25.05)	22.48 (7,555.14)	21.86 (19.73)	4.45 (4.22)
lactic acidosis	301	9.1 (8.12–10.2)	9.06 (2,134.07)	8.97 (8)	3.16 (2.96)
pancreatitis	291	6.41 (5.71–7.2)	6.39 (1,312.65)	6.34 (5.65)	2.67 (2.47)
hypoglycaemia	276	6.32 (5.61–7.11)	6.29 (1,219.46)	6.25 (5.55)	2.64 (2.44)
hyperglycaemia	271	8.24 (7.31–9.29)	8.21 (1,697.91)	8.13 (7.21)	3.02 (2.81)
cellulitis	268	5.29 (4.69–5.96)	5.27 (921.54)	5.24 (4.65)	2.39 (2.19)
thirst	259	14.47 (12.79–16.36)	14.41 (3,172.4)	14.16 (12.52)	3.82 (3.57)
blood glucose decreased	239	5.4 (4.76–6.14)	5.39 (848.54)	5.36 (4.72)	2.42 (2.21)
blood glucose abnormal	230	11.28 (9.9–12.85)	11.24 (2,115.13)	11.09 (9.73)	3.47 (3.22)
dysuria	218	6.09 (5.33–6.96)	6.07 (916.12)	6.03 (5.28)	2.59 (2.36)
gangrene	211	37.47 (32.62–43.03)	37.35 (7,109.95)	35.62 (31.01)	5.15 (4.73)
necrotising fasciitis	210	64.3 (55.83–74.04)	64.09 (12,014.78)	59.12 (51.34)	5.89 (5.33)
abnormal loss of weight	195	26.32 (22.81–30.37)	26.24 (4,575.24)	25.39 (22.01)	4.67 (4.29)
blood creatinine increased	188	3.17 (2.75–3.66)	3.16 (277.48)	3.16 (2.73)	1.66 (1.43)
polyuria	176	22.44 (19.31–26.08)	22.38 (3,491.55)	21.76 (18.73)	4.44 (4.06)
urosepsis	168	18.4 (15.78–21.44)	18.35 (2,690.62)	17.94 (15.39)	4.16 (3.8)

### 3.4 Stratified analysis by individual SGLT-2 inhibitor agent

To evaluate potential heterogeneity in the adverse event signal profiles across different SGLT-2 inhibitors when combined with metformin, we performed a stratified analysis disaggregating the combination cohort by the four constituent agents—canagliflozin, dapagliflozin, empagliflozin, and ertugliflozin. For each agent-specific combination, we calculated the RORs with 95% confidence intervals for the top 10 most frequently reported PTs (**Table 7**). The stratified analysis revealed notable agent-specific signal patterns. In the canagliflozin-plus-metformin subgroup, amputation-related events—exemplified by *toe amputation* (ROR = 234.35, 95% CI: 209.35–262.35)—exhibited markedly higher signal intensities than those observed with the other three SGLT-2i agents. In contrast, *diabetic ketoacidosis* was the most frequently reported PT in both the dapagliflozin and empagliflozin combination subgroups. For the ertugliflozin combination subgroup, the total number of reports was considerably smaller (n = 230), and accordingly, the ROR estimates for individual PTs were accompanied by wide confidence intervals, precluding robust comparative inferences. Collectively, these stratified findings suggest that the adverse event profiles of SGLT-2i plus metformin dual therapy are not monolithic but rather exhibit drug-specific heterogeneity, indicating that pooled aggregate analyses may obscure signal differences that are clinically and pharmacologically relevant.

**Table 7:** Top 10 PTs by reporting frequency and corresponding ROR (95% CI) following concomitant use of four SGLT-2 inhibitors with metformin.

Ertugliflozin + metformin			Dapagliflozin + metformin		
PT	N	ROR (95% CI)	PT	N	ROR (95% CI)
diabetic ketoacidosis	20	85.87 (54.97–134.13)	diabetic ketoacidosis	883	110.15 (102.82–118.00)
blood glucose increased	17	10.16 (6.27–16.46)	weight decreased	436	4.6 (4.19–5.06)
nausea	14	1.92 (1.13–3.26)	blood glucose increased	396	6.47 (5.86–7.15)
dizziness	13	2.85 (1.65–4.94)	nausea	305	1.14 (1.02–1.28)
urine ketone body present	12	858.44 (483.3–1,524.76)	ketoacidosis	296	129.4 (115.01–145.6)
fungal infection	10	30.67 (16.42–57.32)	urinary tract infection	275	4.55 (4.04–5.12)
ketoacidosis	8	120.16 (59.78–241.53)	euglycaemic diabetic ketoacidosis	264	175.22 (154.48–198.74)
urinary tract infection	8	4.8 (2.39–9.64)	vomiting	260	1.67 (1.48–1.89)
vomiting	7	1.63 (0.78–3.44)	acute kidney injury	250	3.92 (3.46–4.44)
weight decreased	7	2.65 (1.26–5.59)	diarrhoea	248	1.07 (0.94–1.21)
Empagliflozin + metformin			Canagliflozin + metformin		
PT	N	ROR (95% CI)	PT	N	ROR (95% CI)
diabetic ketoacidosis	1,369	159.76 (150.99–169.03)	diabetic ketoacidosis	1,250	201.92 (190.29–214.26)
euglycaemic diabetic ketoacidosis	696	482.53 (443.84–524.59)	acute kidney injury	641	12.71 (11.75–13.76)
ketoacidosis	553	230.12 (210.55–251.5)	weight decreased	390	5.07 (4.59–5.61)
vomiting	510	2.97 (2.72–3.24)	osteomyelitis	341	74.81 (67.11–83.39)
blood glucose increased	506	7.44 (6.81–8.12)	toe amputation	333	234.35 (209.35–262.35)
nausea	487	1.64 (1.5–1.8)	blood glucose increased	307	6.16 (5.5–6.9)
weight decreased	461	4.36 (3.97–4.78)	fungal infection	279	29.26 (25.98–32.95)
diarrhoea	351	1.36 (1.22–1.51)	urinary tract infection	252	5.13 (4.53–5.81)
dizziness	347	1.87 (1.68–2.08)	nausea	249	1.14 (1.01–1.3)
fungal infection	341	26.07 (23.42–29.04)	dizziness	222	1.64 (1.43–1.87)

### 3.5 Aggregated analysis of clinically relevant event categories

To complement the PT-level findings and mitigate the fragmentation inherent to highly granular MedDRA terminology, we performed a supplementary aggregated analysis for three prespecified clinically relevant event categories—ketoacidosis events, genital infections, and renal injury events. The pooled results are summarised in **Table 8**. Following aggregation, *ketoacidosis events* constituted the most prominent category within the combination cohort, with 5,653 reported cases and a pooled ROR of 200.36. *Genital infection events* accounted for 1,372 reports (pooled ROR = 45.28), and *renal injury events* for 1,969 reports (pooled ROR = 3.04). This aggregated analytical approach not only corroborated the core safety signals identified at the PT level but also served to consolidate fragmented terms into clinically interpretable endpoints, thereby enhancing the translational relevance of the pharmacovigilance findings.

**Table 8:** ROR analysis of summarised PTs for three major clinical events with concomitant medications.

Event	Number of included PTs	Concomitant medication N	ROR (95% CI)	Positive signal
Ketoacidosis event	6	5,653	200.36 (194.40–206.50)	Yes
Genital infection event	51	1,372	45.28 (42.86–47.85)	Yes
Renal injury event	35	1,969	3.04 (2.90–3.18)	Yes

### 3.6 Sensitivity analysis

To assess the robustness of our primary findings against potential confounding by the background comparator, we performed a prespecified sensitivity analysis restricted to a control population comprising reports involving other antidiabetic agents (excluding both SGLT-2 inhibitors and metformin). The results from this sensitivity framework are presented in the Supplementary Materials and summarised as follows. Signals for *euglycaemic diabetic ketoacidosis* (ROR = 371.58), *Fournier’s gangrene* (ROR = 250.34), *fungal infections* (ROR = 16.07), and *metabolic acidosis* (ROR = 15.88) remained largely consistent in magnitude with those in the primary analyses, indicating that the associations between these adverse events and the SGLT-2i/metformin combination are robust to changes in the reference population. Notably, the ROR for *lactic acidosis* exhibited a marked increase from 9.10 in the primary analysis to 42.71 under the comparator-restricted setting. This elevation suggests that the original estimate may have been attenuated by the inherently high background incidence of lactic acidosis among metformin users, potentially leading to an underestimation of the true risk attributable to the combination regimen. Conversely, signals for *weight decreased*, *blood glucose increased/decreased*, *glycated haemoglobin increased*, and *blood creatinine increased* were substantially attenuated or dropped out of the top-ranked signals in the sensitivity analysis, implying that these findings may plausibly reflect the underlying diabetic disease state or general glycaemic control effects common to most glucose-lowering therapies, rather than a specific safety liability unique to the SGLT-2i/metformin dual therapy (**Supplementary Table 2**).

### 3.7 Time-to-onset analysis

**Figure 5:**
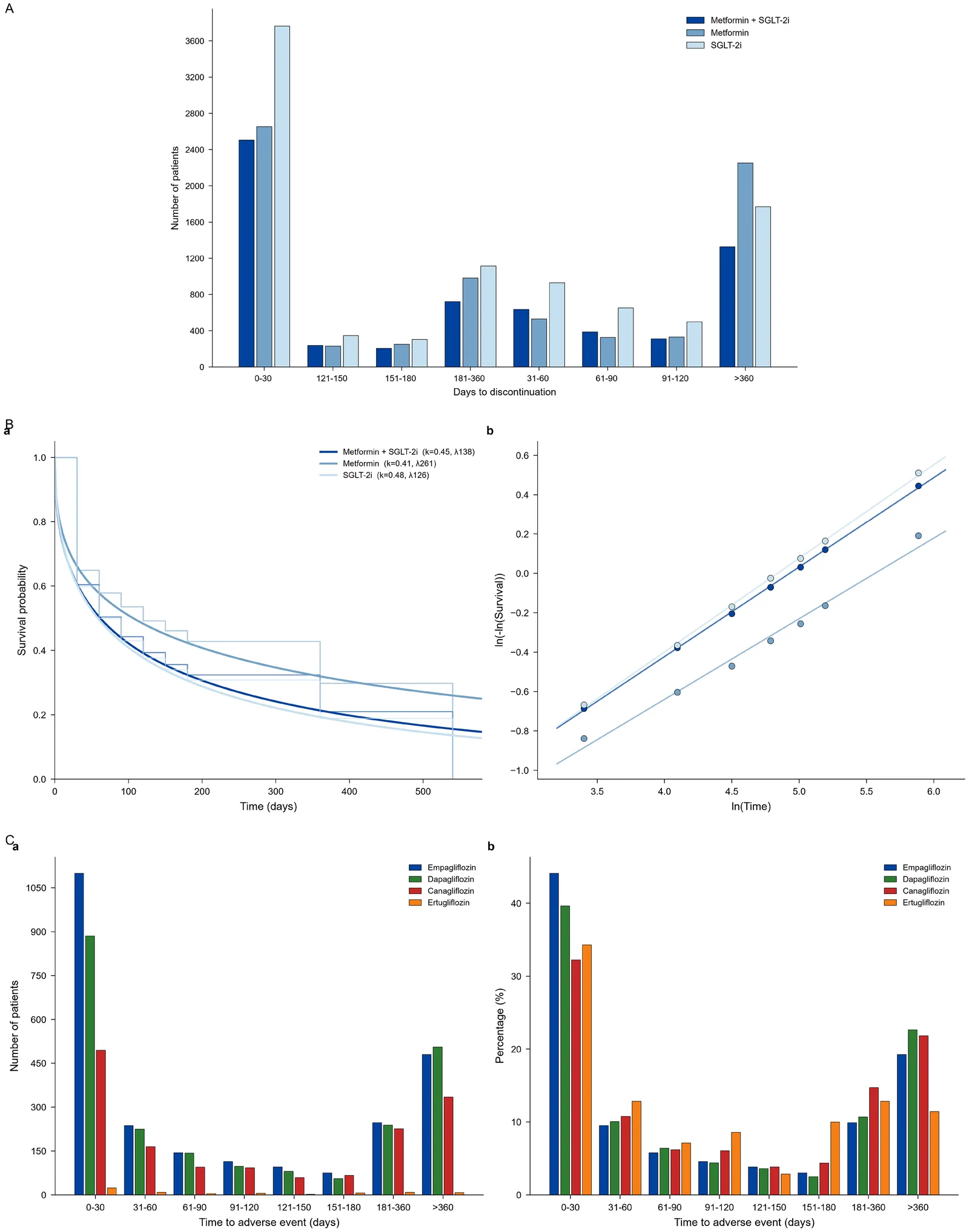
Time-to-onset distribution of adverse events across the three treatment regimens. [**(A)** Number of adverse reactions occurring in different time periods; **(B)** Schematic diagram of Weibull fitting; **(C)** Number and percentage of adverse reaction reports for four different SGLT-2 inhibitors across various time periods].

Of the 23,407 reports in the combination cohort, 17,075 (72.95%) were excluded from the time-to-onset analysis owing to missing treatment start dates (START_DT) or adverse event occurrence dates (EVENT_DT), chronological inconsistencies (*EVENTDT* < *STARTDT*), or imprecise date records. This yielded a final analytical subset of 6,332 reports (27.05% of the total combination reports). Within this evaluable cohort, 2,505 adverse events (39.56%) occurred within the first 30 days of treatment initiation, whereas 1,329 events (20.99%) occurred beyond 360 days (**Figure 5A**). To characterise the latency distribution of target adverse events across the three exposure groups, we fitted a Weibull parametric survival model to the time-to-event data. The Weibull probability plots (**Figure 5B**) demonstrated that the empirical scatter points for all three groups were closely aligned with the fitted linear reference lines, thereby validating the assumption of a Weibull distribution and confirming the reliability of the model fit. The estimated shape parameters (k) were 0.41 for the metformin monotherapy group, 0.45 for the combination group, and 0.48 for the SGLT-2i monotherapy group. Notably, k was consistently < 1 across all three cohorts, with the upper bounds of the corresponding 95% confidence intervals remaining below unity, indicating that the hazard of adverse events was highest early during the treatment course and declined progressively with extended follow-up time. The estimated survival curves further revealed that, at any given follow-up time point, the SGLT-2i monotherapy group exhibited the lowest survival probability (i.e., the shortest time to event occurrence), whereas the metformin monotherapy group had the highest survival probability (i.e., the longest latency). The combination group demonstrated an intermediate time-to-onset profile, positioned between the two monotherapy arms. The distribution differences in the number of adverse event reports within 30 days among different SGLT‑2i agents are detailed in **Figure 5C**. Among the four specific SGLT‑2i drugs, empagliflozin had the highest number of reports within 30 days, followed by dapagliflozin, and the proportion pattern was consistent with this ranking.

### 3.8 Comparative PT-level signal profiles across the three exposure cohorts

**Figure 6:**
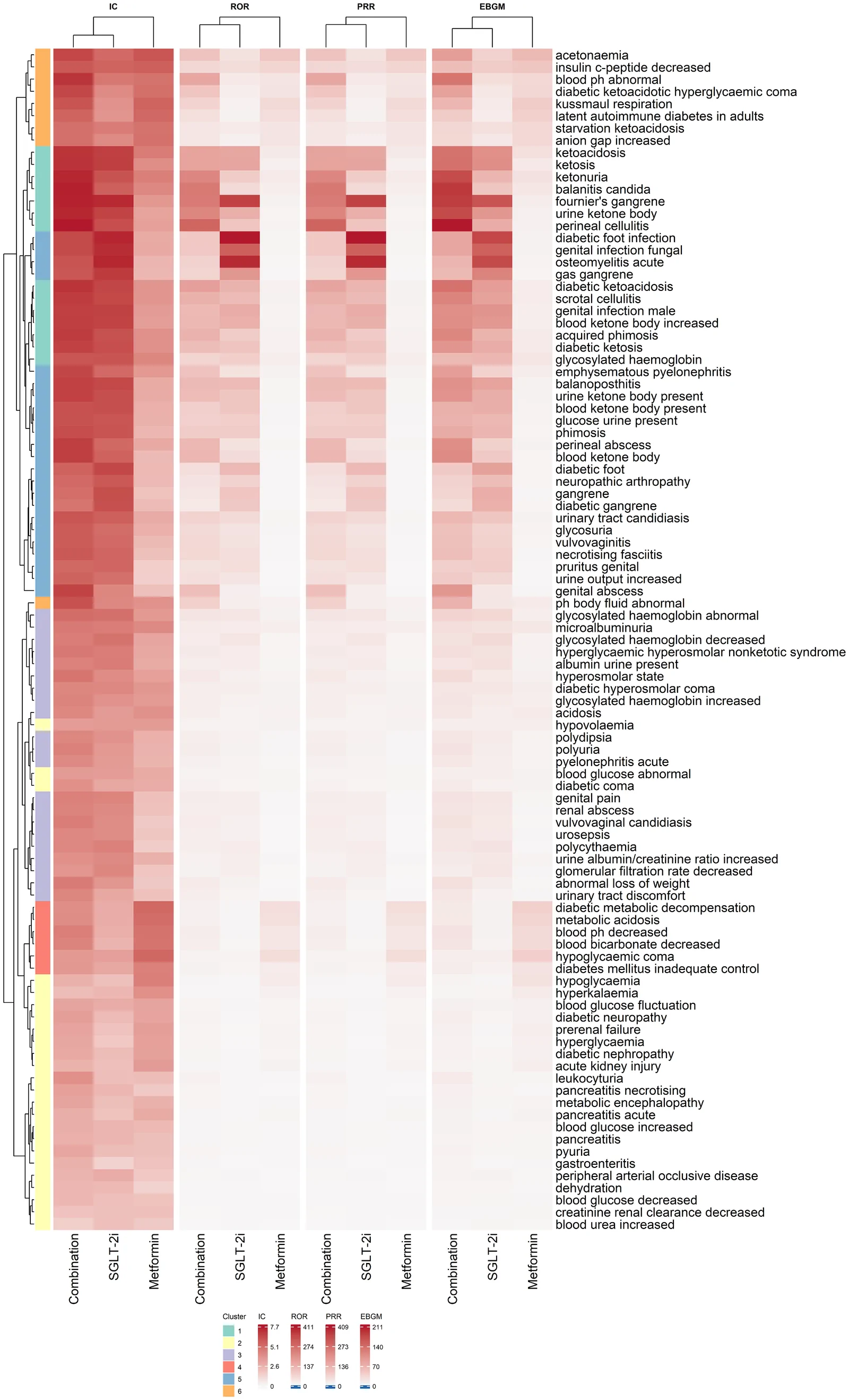
Heatmap of signal intensity at the Preferred Term (PT) level across the three treatment regimens (based on ROR, PRR, EBGM, and IC methods).

To visually dissect the differential signal patterns across the three exposure regimens, we performed a heatmap analysis based on adverse events that simultaneously satisfied two criteria: (1) ranking among the top 100 most frequently reported PTs in the combination cohort, and (2) meeting the prespecified positive signal thresholds for all four disproportionality algorithms. As illustrated in **Figure 6**, the signal intensity profile of the combination regimen was broadly consistent with that of SGLT-2i monotherapy, whereas it diverged markedly from that of metformin monotherapy. Within the combination cohort, the top five PTs ranked by signal intensity across all four algorithms were consistently identified as perineal cellulitis, candidal balanitis, Fournier’s gangrene, urinary ketone bodies, and ketonuria, underscoring a signal signature predominantly driven by metabolic and infectious/infestation categories closely aligned with the known pharmacological effects of SGLT-2 inhibition.

### 3.9 Clinical outcomes

**Figure 7:**
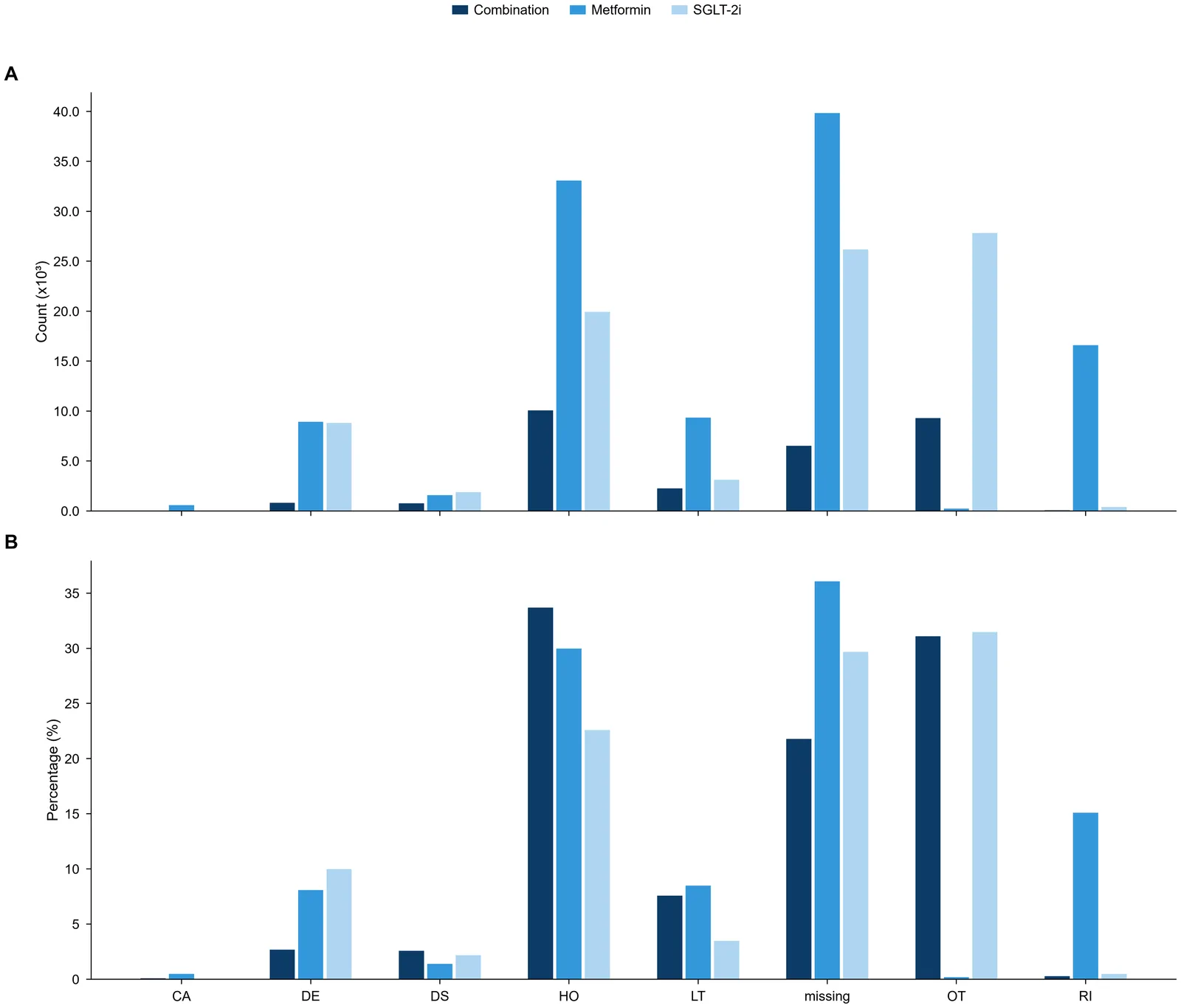
Number and percentage of serious outcome reports by medication regimen. **(A)** Number of serious outcome reports; **(B)** Percentage of serious outcome reports. Abbreviations: CA, Congenital Anomaly; DE, Death; DS, Disability; HO, Hospitalization – Initial or Prolonged; LT, Life-Threatening; OT, Other Serious (Important Medical Event); RI, Required Intervention to Prevent Permanent Impairment/Damage.

Among the 23,407 reports in the combination cohort, a total of 29,931 clinical outcome entries were documented, reflecting that individual reports could record multiple concurrent outcomes. The distribution of these outcomes was as follows: *hospitalisation or prolonged hospitalisation* (n = 10,083, 33.7%), *other serious medical events* (n = 9,321, 31.1%), *life-threatening events* (n = 2,286, 7.6%), *death* (n = 821, 2.7%), *disability* (n = 786, 2.6%), *required intervention to prevent permanent impairment/damage* (n = 75, 0.3%), *congenital anomaly* (n = 23, 0.1%), and *missing outcome data* (n = 6,536, 21.8%). As depicted in **Figure 7**, the distribution of serious adverse outcomes observed with SGLT-2i exposure was broadly comparable to that of the combination therapy. Notably, the proportion of reports with congenital anomalies was higher in the metformin monotherapy group, a pattern that diverges from the other two exposure cohorts and warrants further attention in subsequent safety assessments.

## 4 Discussion

In the clinical management of complex or refractory diseases, combination pharmacotherapy is not merely commonplace but often clinically unavoidable. While such regimens may confer synergistic efficacy and expanded therapeutic coverage, they also carry the potential for increased adverse event liabilities (22). Diabetes mellitus, as a chronic metabolic disorder necessitating long-term disease management, frequently renders monotherapy insufficient for sustaining durable glycaemic control; consequently, combination strategies have become a cornerstone of routine clinical practice. With the global prevalence of diabetes continuing its upward trajectory, the population exposed to multidrug regimens is concomitantly expanding. Leveraging real-world data from the FAERS database spanning the first quarter of 2013 through the fourth quarter of 2025, we systematically profiled the adverse event signal landscape associated with the SGLT-2i and metformin dual regimen. Our analysis incorporated 23,407 combination-related adverse event reports, distributed across 27 distinct System Organ Classes (SOCs). At the SOC level, ROR-based signal detection revealed moderate-to-strong signal intensities for *metabolism and nutrition disorders* (ROR = 9.00), *renal and urinary disorders* (ROR = 3.91), *reproductive system and breast disorders* (ROR = 2.59), and *infections and infestations* (ROR = 2.49). In contrast, although *cardiac disorders* (ROR = 1.20) and *gastrointestinal disorders* (ROR = 1.15) marginally surpassed the prespecified detection threshold, the corresponding signal intensities were notably weak, and at the PT level, none of the individual events within these two SOCs consistently met the simultaneous four-algorithm criteria for positive signals, effectively yielding a null signal upon stringent validation. Therefore, the following discussion centres on the three SOC categories that exhibited both robust signal intensities and substantial reporting volumes—namely, *metabolism and nutrition disorders*, *renal and urinary disorders*, and *infections and infestations*—as these represent the most clinically salient findings from the present pharmacovigilance investigation.

### 4.1 Baseline demographic and temporal characteristics

In the combination cohort, the sex distribution showed a marginal predominance of male patients (51.3% male versus 44.5% female). This pattern likely reflects a confluence of factors beyond true sex-specific risk, including reporting biases inherent in the FAERS spontaneous reporting system, differential prescribing patterns of individual SGLT-2 inhibitors across sexes, and sex-related disparities in healthcare access or reporting behaviour. Regarding temporal trends, adverse event reports for the combination regimen were recorded in FAERS as early as 2013, with a progressive increase in reporting volume observed from 2014 onwards. This trajectory aligns closely with the clinical introduction and real-world use of SGLT-2 inhibitors—the first agent in this class, dapagliflozin, received European marketing authorisation in late 2012 and was subsequently adopted into routine practice over the ensuing years (23, 24). The close correspondence between our data trends and the known clinical deployment timeline supports the external validity of the present FAERS-based pharmacovigilance snapshot.

### 4.2 Predominant adverse event signals: focus on ketoacidosis

Among the SOCs with positive signals, *metabolism and nutrition disorders* emerged as the category with the highest signal intensity, within which diabetic ketoacidosis (DKA) and euglycaemic diabetic ketoacidosis (euDKA) constituted the predominant PT-level events. Notably, the ROR point estimate for euDKA in the combination group (335.43) was numerically higher than that observed in the SGLT-2i monotherapy group (185.24), suggesting a potential augmentation of this specific risk when metformin is added to SGLT-2i therapy. At the individual PT level, DKA was the single most frequently reported term (n=3,522). However, following the prespecified aggregation of related terms—including DKA, euDKA, ketoacidosis, ketosis, and ketonuria—the composite *ketoacidosis events* category encompassed a total of 5,653 reports, accounting for approximately 24.15% of all combination-related reports. This aggregated figure substantially exceeds the count of any individual PT and underscores that the SGLT-2i/metformin combination is most prominently characterised by a heightened signal for ketoacidosis-related events, warranting particular vigilance in routine clinical practice.

The ketogenic propensity of SGLT-2 inhibitors is mechanistically rooted in their action on the renal proximal tubule: by inhibiting glucose reabsorption, these agents markedly increase urinary glucose excretion, thereby lowering plasma glucose without raising insulin levels. This is accompanied by a compensatory rise in glucagon concentration, which elevates the glucagon-to-insulin ratio and consequently promotes hepatic ketogenesis (25). Indeed, multiple studies have demonstrated that SGLT-2i therapy is associated with increased serum ketone body levels, most notably β-hydroxybutyrate (26). Interestingly, one previous report suggested that the SGLT-2i-induced elevation in β-hydroxybutyrate was attenuated when metformin was co-administered, potentially through metformin’s suppression of gluconeogenesis, which may partially offset the ketogenic stimulus (27). This prior observation, however, stands in apparent contrast to our real-world pharmacovigilance findings, which revealed a descriptively higher euDKA reporting signal for the combination regimen than for SGLT-2i monotherapy. Several non-mutually exclusive explanations may account for this discrepancy. First, *indication bias*—patients receiving combination therapy may have a longer diabetes duration, more compromised β-cell function, or concurrent insulin use, all of which are established independent risk factors for euDKA. Second, *detection bias*—clinicians may maintain a heightened clinical awareness of euDKA in patients on combination therapy, thereby increasing the likelihood of reporting. Third, *residual confounding*—the FAERS database lacks detailed information on key precipitating factors for ketoacidosis, such as low-carbohydrate diets, acute intercurrent infections, or surgical stress, all of which may act as triggers in susceptible individuals. Collectively, these considerations underscore a critical caveat: our findings indicate a signal enrichment for euDKA in reports involving the combination therapy, but they do not establish that metformin potentiates the ketogenic effect of SGLT-2 inhibition. Definitive elucidation of this causal question will require prospective active-surveillance cohort studies with rigorous adjustment for the aforementioned confounders.

The positive signal observed for the *renal and urinary disorders* SOC in the combination group (ROR = 3.91) appears, on the surface, to conflict with the well-established renoprotective profile of SGLT-2 inhibitors reported extensively in the literature (28, 29). Indeed, multiple large-scale randomised controlled trials and meta-analyses have demonstrated that SGLT-2 inhibitors significantly reduce the risk of acute kidney injury (AKI) (relative risk [RR] = 0.77, 95% CI: 0.70–0.84) (30), attenuate the progression of end-stage kidney disease, and lower cardiovascular mortality (31, 32). Several non-mutually exclusive considerations may help reconcile this apparent discrepancy. First, adverse renal events reported in FAERS should not be equated with drug-induced nephrotoxicity *per se*; rather, they may be confounded by underlying renal impairment at baseline, concomitant use of nephrotoxic agents (e.g., diuretics, non-steroidal anti-inflammatory drugs, or contrast media), or intercurrent conditions that predispose to renal dysfunction. Second, although metformin is not intrinsically nephrotoxic, it is predominantly excreted renally, and its accumulation in the setting of reduced glomerular filtration may increase the risk of lactic acidosis (33). Furthermore, while some animal studies have suggested that high-dose metformin may exacerbate renal tubular epithelial injury (34), the clinical translatability of these preclinical findings remains uncertain. Collectively, the signal enrichment observed in the renal SOC for the combination regimen may more plausibly reflect the underlying burden of preexisting renal disease in this patient population, or the influence of concomitant medications, rather than a direct nephrotoxic effect of the combined therapy *per se*. From a clinical standpoint, the SGLT-2i/metformin combination remains a safe and guideline-recommended therapeutic option for patients with preserved renal function; nevertheless, routine monitoring of renal function before initiation and at regular intervals during treatment is judiciously advised.

Infectious adverse events also emerged as a prominent safety concern in the context of SGLT-2i therapy, whether administered as monotherapy or in combination. One study comparing SGLT-2 inhibitors with metformin as initial therapy reported that the risk of genital infections among SGLT-2i users was substantially elevated (HR = 2.28, 95% CI: 1.87–2.78) (35); a large-scale cohort study yielded closely comparable findings (HR = 2.19, 95% CI: 1.91–2.51) (36). Mechanistically, SGLT-2 inhibitors increase urinary glucose concentrations by blocking renal tubular glucose reabsorption, thereby creating a nutrient-rich microenvironment that facilitates the proliferation of pathogenic microorganisms—notably *Candida* species—and consequently predisposes to genitourinary tract infections (37). With respect to the combination regimen specifically, the infectious adverse events observed are largely attributable to the SGLT-2 inhibitor component *per se*, manifesting as a markedly elevated risk of genital infections and a moderate increase in the risk of urinary tract infections.

Weight loss also emerged as a notable adverse event in the combination cohort, ranking second in reporting frequency, and thus warrants brief consideration here. Both SGLT-2 inhibitors and metformin, when used as monotherapy, have been independently associated with weight reduction. A systematic review and network meta-analysis reported that SGLT-2i monotherapy yielded a mean weight reduction of –3.20 kg (95% CI: –4.69 to –1.72), whereas metformin monotherapy resulted in a more modest reduction of –1.93 kg (95% CI: –3.01 to –0.85) (38). Similarly, a separate meta-analysis focusing on non-diabetic overweight or obese individuals showed that SGLT-2i monotherapy for more than 12 weeks led to significant weight loss relative to placebo (–2.32 kg versus –1.01 kg) (39). Mechanistically, the weight-lowering effect of SGLT-2 inhibitors is primarily driven by the induction of a negative energy balance through increased urinary glucose excretion, which in turn yields moderate reductions in body weight and may improve body composition—including a decrease in visceral adipose tissue (40). Additional evidence suggests that SGLT-2 inhibitors may modulate body weight through alterations in fat mass or fluid distribution, and may influence adipose tissue metabolism by promoting white adipose tissue “browning” and enhancing fatty acid oxidation (41, 42). In contrast, metformin-induced weight loss is at least partially mediated by the upregulation of growth differentiation factor 15 (GDF15), which suppresses appetite and thereby reduces caloric intake (43).

### 4.3 Stratified analysis by individual SGLT-2 inhibitor agents

The stratified analysis revealed substantial heterogeneity in the adverse event signal profiles across individual SGLT-2 inhibitors when combined with metformin. Notably, amputation-related signals in the canagliflozin combination subgroup were markedly higher than those observed with the other three agents, a finding that aligns with previous regulatory warnings and published evidence specifically linking canagliflozin to an increased risk of lower-extremity amputation (44). In the dapagliflozin and empagliflozin combination subgroups, diabetic ketoacidosis (DKA) was the most frequently reported PT, a pattern that may plausibly be attributed to the broader real-world prescribing volume of these two agents—dapagliflozin, in particular, being the first-in-class SGLT-2 inhibitor to receive marketing authorisation and consequently holding the largest market share. For the ertugliflozin subgroup, the wide confidence intervals surrounding the ROR estimates for individual PTs precluded robust comparative inferences, reflecting the limited number of reports available for this agent (n = 230). Collectively, these stratified findings carry an important methodological implication: pooling all four SGLT-2 inhibitors into a single aggregate analysis may average out agent-specific signal differences, thereby diluting signals that are clinically meaningful. The amputation signal for canagliflozin, for instance, was notably attenuated in the combined estimates. Therefore, for adverse events with established or suspected agent-specific predispositions—such as amputation or ketoacidosis—priority should be given to agent-stratified estimates rather than pooled summary measures when interpreting safety signals in clinical practice.

### 4.4 Time-to-onset analysis and interpretative caveats

Adverse events emerging during glucose-lowering therapy frequently manifest early in the treatment course. For instance, in studies of danuglipron (a glucagon-like peptide-1 [GLP-1] receptor agonist), nausea, vomiting, abdominal pain, and diarrhoea were among the most common early-onset events, predominantly mild to moderate in severity (45). Similarly, gastrointestinal adverse events—when assessed as an endpoint—have been reported to occur within the first 1–4 weeks of treatment initiation (46). In the present study, 39.56% of adverse events in the evaluable time-to-onset subset occurred within the first 30 days, suggesting that the early treatment phase constitutes a critical window for safety monitoring. Concurrently, however, 20.99% of events occurred beyond 360 days, underscoring that the risk is not confined to the initial period but persists over the longer term. To further characterise the temporal dynamics of event risk, we fitted Weibull distributions to the time-to-event data for each of the three cohorts. The estimated shape parameters (k) were 0.41, 0.45, and 0.48 for the metformin monotherapy, combination, and SGLT-2i monotherapy groups, respectively—all below unity—indicating that the hazard of adverse events was highest early after treatment initiation and declined progressively thereafter.

A critical caveat must be acknowledged regarding the time-to-onset analysis: the FAERS database suffers from substantial missingness in temporal variables. Of the 23,407 combination-group reports, 72.95% (17,075) were excluded from this analysis owing to missing treatment start dates (START_DT) or event occurrence dates (EVENT_DT), leaving only 27.05% of reports with evaluable time data. Importantly, this high exclusion rate is unlikely to be missing completely at random; rather, it reflects a systematic selection bias. Previous studies have demonstrated that reports with complete date information in FAERS are more likely to originate from healthcare professionals (e.g., physicians or nurses) and are more frequently associated with serious outcomes such as hospitalisation or life-threatening events (47). This non-random missingness pattern has direct implications for interpretation: the observed proportion of 39.56% of events occurring within 30 days may overestimate the true early-event rate in the entire combination-treated population. Conversely, mild, chronic, or non-hospitalised events—which are less likely to be precisely dated and promptly reported—are disproportionately excluded, potentially biasing the analysed subset towards more acute and severe presentations. Consequently, our findings should be interpreted specifically as indicating that, *within the subset of serious or professionally reported events for which precise dates are available*, a substantial proportion occurred early in the treatment course; they should not be generalised to imply that 39.56% of all events in the overall combination cohort occur within 30 days. To partially mitigate the impact of this censoring and missingness, we employed a Weibull parametric model, which exhibits a degree of robustness to data imperfections. Nevertheless, future active surveillance studies leveraging real-world electronic health records (EHRs) with complete and systematically captured treatment timelines will be essential to provide a more accurate and unbiased characterisation of the temporal safety profile of this combination regimen.

### 4.5 Clinical outcomes and serious adverse event profile

We further examined the distribution of serious clinical outcomes associated with the SGLT-2i/metformin combination. Among reports with documented outcomes, *hospitalisation or prolonged hospitalisation* constituted the most frequent category (33.7%), followed by *other serious medical events* (31.1%) and *life-threatening events* (7.6%). The overall outcome distribution was broadly comparable between the combination and monotherapy cohorts, suggesting that the addition of metformin to SGLT-2i therapy does not introduce an excess risk of fatal outcomes. The principal identifiable causes of hospitalisation in the combination group included diabetic ketoacidosis (DKA) and euglycaemic diabetic ketoacidosis (euDKA)—with 3,522 and 1,114 reports, respectively—alongside severe infections (e.g., Fournier’s gangrene, n = 556; sepsis, n = 459), acute kidney injury (n = 1,153), and serious gastrointestinal reactions. Of particular clinical concern, the signal intensity for euDKA in the combination group (ROR = 335.43) substantially exceeded that observed in the SGLT-2i monotherapy group. Given that euDKA presents with normal or only mildly elevated blood glucose levels, it is prone to delayed recognition or misdiagnosis and, if not promptly treated, can rapidly evolve into a life-threatening emergency (48). Therefore, for patients receiving this combination regimen, the presence of non-specific symptoms—including nausea, vomiting, abdominal pain, dyspnoea, or malaise—should prompt immediate measurement of blood ketone levels and arterial blood gas analysis, even when plasma glucose appears within the normal range. Despite the potential for serious outcomes—among which hospitalisation predominates—the proportions of life-threatening events and death remained relatively low in the present pharmacovigilance dataset. In clinical practice, a comprehensive risk-mitigation strategy encompassing rigorous patient selection, early recognition of adverse events, and structured patient education is essential to minimise the incidence of these severe complications.

This study provides a comprehensive pharmacovigilance survey of safety signals associated with the SGLT-2i and metformin combination, drawing on data from the U.S. FDA Adverse Event Reporting System (FAERS). While our findings offer clinically relevant insights, their interpretation must be tempered by several inherent limitations of the FAERS database and our analytical approach. First, as a spontaneous reporting system, FAERS is subject to underreporting, selective reporting, and reporting bias. Mild or common events, or events with uncertain causal relationships—such as minor gastrointestinal symptoms—are likely to be under-represented, whereas severe, rare, or novel events—including euDKA and Fournier’s gangrene—tend to be disproportionately reported, potentially inflating their apparent signal intensities. This phenomenon plausibly accounts, at least in part, for the exceptionally high ROR estimates observed for these rare events in our study (335.43 and 249.81, respectively). Second, at the statistical level, we performed signal detection across 27 SOCs and hundreds of PTs, entailing a substantial multiplicity of hypothesis tests. Although we implemented a stringent four-algorithm consensus threshold—namely, ROR 95% CI lower bound > 1, PRR ≥ 2 with χ² ≥ 4, IC025 > 0, and EBGM05 > 2—to mitigate the risk of false-positive findings, the potential for type I error inflation due to multiple comparisons cannot be entirely excluded. Signals that fell marginally above the detection threshold (e.g., SOC-level signals with ROR lower bounds only slightly exceeding unity) may represent chance findings. Conversely, certain rare adverse events may have escaped detection owing to insufficient statistical power resulting from very low report counts, raising the possibility of type II errors (false negatives). Accordingly, all positive signals reported herein should be interpreted as hypothesis-generating and exploratory in nature, warranting independent validation in future studies. Third, substantial proportions of reports suffered from missing data, including body weight (60.8%) and age (23.9%). Moreover, the FAERS database lacks critical clinical covariates essential for rigorous confounding control, including but not limited to diabetes type (type 1 versus type 2), pancreatic β-cell functional status, concomitant medications (e.g., diuretics, renin–angiotensin–aldosterone system [RAAS] blockers, or insulin dose adjustments), recent surgery or fasting status, intercurrent infections, and baseline renal function. Many of these factors are established risk determinants for DKA, euDKA, and acute kidney injury, and their absence from the database precluded adjusted sensitivity analyses, potentially introducing residual confounding into our estimates. Fourth, regarding analytical strategy, although we performed agent-stratified analyses and identified inter-drug signal heterogeneity, the results for the ertugliflozin subgroup should be interpreted with particular caution owing to the small sample size (n = 230), which yielded imprecise estimates with wide confidence intervals. In addition, for reports involving multiple SGLT-2 inhibitors, our stratification approach counted such reports in each relevant agent subgroup, which may introduce a minor degree of duplicate-counting bias. Furthermore, it is important to emphasise that disproportionality analyses—whether ROR, PRR, BCPNN, or MGPS—can only establish statistical associations between a drug and an adverse event; they cannot demonstrate causation. Notably, we did not employ specialised drug–drug interaction signal detection methods such as the Ω shrinkage measure; consequently, our comparative observations between the combination and monotherapy groups are restricted to descriptive differences in signal intensities and should not be construed as evidence that the combination confers an incremental safety risk beyond that attributable to its individual components.

In this study, we leveraged real-world data from the FAERS database and applied a multi-model disproportionality analysis framework to systematically profile the adverse event reporting landscape associated with the SGLT-2 inhibitor and metformin combination in routine clinical use. Our findings indicate that statistically enriched signals were primarily concentrated in three System Organ Class categories—*metabolism and nutrition disorders*, *renal and urinary disorders*, and *infections and infestations*. Stratified analyses further revealed heterogeneity in the signal profiles across individual SGLT-2 agents when combined with metformin, with particular attention warranted for amputation-related signals uniquely prominent in the canagliflozin combination subgroup. Of notable clinical and pharmacological interest, the signal intensity for euglycaemic diabetic ketoacidosis (euDKA) ranked among the highest of all detected signals (ROR = 335.43), and more than one-third of the evaluable AE reports occurred within the first 30 days of treatment initiation. This temporal clustering offers a potentially actionable window for refined pharmacovigilance monitoring and early clinical detection strategies. Nevertheless, owing to the inherent limitations of the FAERS spontaneous reporting system—including underreporting, selective reporting, missing data, and residual confounding—all signals identified in this study remain strictly exploratory statistical findings and do not constitute confirmatory clinical evidence. The principal value of the present work lies in providing a hypothesis-generating source for prioritising specific adverse events—particularly euDKA—for future risk factor screening and prospective study design. Definitive elucidation of the underlying pathophysiology and causal relationships will require integration of mechanistic investigations and large-scale, well-phenotyped longitudinal cohort studies with rigorous confounding control.

## Data Availability

The original contributions presented in the study are included in the article/**Supplementary Material**. Further inquiries can be directed to the corresponding authors.
